# A Comparison of Parent Reports, the Mental Synthesis Evaluation Checklist (MSEC) and the Autism Treatment Evaluation Checklist (ATEC), with the Childhood Autism Rating Scale (CARS)

**DOI:** 10.3390/pediatric16010016

**Published:** 2024-03-11

**Authors:** Rebecca Netson, Andriane Schmiedel Fucks, Andressa Schmiedel Sanches Santos, Lucas Ernesto Pavoski Poloni, Nilson Noboru Nacano, Elielton Fucks, Katarina Radi, William E. Strong, Alice Aparecida Carnaval, María Russo, Rohan Venkatesh, Andrey Vyshedskiy

**Affiliations:** 1 Independent Researcher, Boston, MA 02467, USA; rebecca.netson@gmail.com; 2 Somare Therapeutic and Educational Clinic, Foz do Iguaçu 85851-240, Brazil; andrianesf@gmail.com (A.S.F.); fga.andressa.sml@gmail.com (A.S.S.S.); lucaspoloni@hotmail.com (L.E.P.P.); projetomitasomare@gmail.com (N.N.N.); eligeofoz@gmail.com (E.F.); 3 School of Education, Notre Dame of Maryland University, Baltimore, MD 21210, USA; katarina.radi@bancroft.org; 4 Neato Neuro LLC, Coeur d'Alene, ID 83815, USA; william@neatoneuro.com; 5 Associação Espírita Arco-Íris, Avaré 18705-010, Brazil; alicecarnaval27@gmail.com; 6 Sociedad Venezolana para Niños y Adultos Autistas, Centro de Diagnóstico y Tratamiento para Autismo, Caracas F4XG+7G6, Venezuela; sovenia@gmail.com; 7 ImagiRation LLC, Boston, MA 02135, USA; rohan.t.venki@gmail.com; 8 MET, Boston University, Boston, MA 02215, USA

**Keywords:** MSEC, ATEC, language delay, developmental disorder, autism, psychological evaluations, language therapy, ASD

## Abstract

This study compares two parent reports, the Mental Synthesis Evaluation Checklist (MSEC) and the Autism Treatment Evaluation Checklist (ATEC), with the Childhood Autism Rating Scale (CARS). The ATEC consists of four subscales, as follows: (1) expressive language, (2) sociability, (3) sensory awareness, and (4) health. The MSEC is complementary to the ATEC in measuring complex language comprehension. The parents of 143 autistic children, from 2 to 22 years of age (mean 6.7 ± 5.1 years), completed the MSEC and the ATEC questionnaires and a clinician assessed their CARS score. The CARS score correlated strongly with all parent reports, the complex language comprehension MSEC (*r* = 0.60, *p* < 0.0001), expressive language (*r* = 0.66, *p* < 0.0001), sociability (*r* = 0.58, *p* < 0.0001), sensory awareness (*r* = 0.71, *p* < 0.0001), and health (*r* = 0.53, *p* < 0.0001), as well as the total ATEC score (*r* = 0.75, *p* < 0.0001). The strongest correlation was between the CARS score and the composite of all five parent-reported scores (total ATEC + MSEC, *r* = 0.77, *p* < 0.0001). These results suggest a high fidelity of the MSEC and ATEC parent reports and especially of their composite score, total ATEC + MSEC.

## 1. Introduction

Autism spectrum disorder (ASD) is a neurodevelopmental condition characterized by challenges in social interaction, communication, and repetitive or restricted behaviors [1]. Individuals with ASD may benefit from various treatments and interventions tailored to address their unique needs, including behavioral therapies, educational support, and sometimes medical interventions [2,3,4,5]. An early diagnosis and personalized interventions both play crucial roles in fostering positive outcomes for individuals with ASD [6,7,8]. There is a significant interest in using parent reports to assess the efficacy of ASD treatments [9,10,11,12,13,14,15]. Parents’ assessments improve the scope and granularity of outcome measures with little additional cost. The royalty-free Autism Treatment Evaluation Checklist (ATEC) has been available since 2000 [16]. The ATEC has four subscales, as follows: expressive language, sociability, sensory awareness, and health (Tables S1–S4). Various studies have confirmed the validity and reliability of the ATEC [17,18] and several trials have confirmed the ATEC’s ability to measure longitudinal change in participant performance [19,20,21,22]. Whitehouse and colleagues employed the ATEC as the primary outcome measure in their randomized controlled trial investigating the efficacy of their iPad-centered intervention for ASD, known as TOBY, and noted the ATEC’s “internal consistency and adequate predictive validity” [15].

The ATEC, however, does not measure complex language comprehension, an important indicator of a child’s development. As an example, in one study, the complex language comprehension score was found to be 90% correct at predicting autistic teenagers’ high-functioning versus low-functioning class assignment compared to only 50% accuracy exhibited by their IQ score [23]. In order to measure complex language comprehension in young children, we have developed the royalty-free, 20 item parent report Mental Synthesis Evaluation Checklist (MSEC, Table S5) [24]. Tested with 3715 parents of ASD children, the MSEC demonstrated a good psychometric quality with a strong internal reliability (Cronbach’s alpha > 0.9) [24]. The MSEC exhibited a good construct validity, an adequate test–retest reliability, and a good known-group validity, reflected by contrasts in the MSEC score between children of different ASD severity levels. Another study confirmed the utility of the MSEC in distinguishing the language trajectories in children with ASD (N = 29,138) from those of typically developing children (N = 111) as early as two years of age [25]. The relationship between the MSEC and the ATEC parent reports and ASD severity was investigated in 9573 autistic children [26]. The difference in scores between mild and moderate ASD, as well as between moderate and severe ASD, attained statistical significance in every subscale and in every age group in children from three years of age and older.

Seven recent studies demonstrated that the complex language comprehension score measured using the MSEC and the expressive language subscale score measured using the ATEC provide complementary information (Table 1). (1) In a three-year epidemiological study investigating the effect of passive video and television watching (N = 3227), the complex language scale showed the opposite effect to that measured on the expressive language scale, as follows: shorter video and television watching times were associated with a 1.4-fold (*p* = 0.0128) greater improvement in the complex language comprehension score, but a 1.3-fold (*p* = 0.0719) reduction in the expressive language score [27]. (2) In another three-year study (N = 6454), children who engaged with a therapeutic language intervention increased their complex language comprehension score 2.2-fold, when compared to children with similar initial evaluations (*p* < 0.0001). At the same time, the difference between the groups in the expressive language score was only 1.4-fold (*p* = 0.0144) [28]. (3) In a three-year study on the effect of pretend play (N = 7069), pretend play was associated with a 1.9-fold faster improvement of complex language comprehension (*p* < 0.0001), but only a 1.4-fold faster improvement of expressive language (*p* < 0.0001) [29]. (4) In a three-year study of diet and food consumption (N = 5553), a gluten-free diet was associated with a 1.5-fold faster improvement of complex language comprehension (*p* < 0.0001), but no significant improvement of expressive language (*p* = 0.5918) [30]. (5) In the same study, meat and egg consumption was associated with a 1.6-fold faster improvement of complex language comprehension (*p* < 0.0001), but only a 1.1-fold faster improvement of expressive language (*p* = 0. 0279) [30]. (6) Also in the same study, vegetable consumption was associated with a 1.5-fold faster improvement of complex language comprehension (*p* < 0.0001), but only a 1.2-fold faster improvement of expressive language (*p* = 0. 0137) [30]. (7) And finally, again in the same study, raw-vegetable consumption was associated with a 1.5-fold faster improvement of complex language comprehension (*p* < 0.0001), but no significant improvement of expressive language (*p* = 0. 2307) [30].

This dissociation between receptive and expressive language trajectories observed in these seven studies strongly demonstrates the benefit of a regular complex language comprehension assessment in addition to expressive language evaluations. Put simply, if the complex language comprehension data were not available in the television study [27], the study conclusion would have been that a longer television watching time merely had a positive effect on children with ASD, as exhibited by the expressive language subscale. Only with the addition of the complex language comprehension scale was it possible to detect the significant negative effect of passive video and television watching. Moreover, if the complex language comprehension scale was not available, it would not be possible to detect the positive effect on language development of the gluten-free diet and raw-vegetable-eating, since the expressive language scale did not show any statistically significant effect of the diet [30].

While the MSEC and ATEC can improve treatment efficacy measurements in clinical trials at little additional cost, they remain largely obscure to most researchers. To increase acceptance of the MSEC and ATEC, this study aims to compare the MSEC and ATEC to a well-accepted instrument for measuring ASD severity, the Childhood Autism Rating Scale (CARS) [31]. The ATEC, but not the MSEC, was previously compared to the CARS. Geier et al. studied 56 autistic children (2–16 years of age) and reported a strong correlation between the CARS score and the total ATEC score (*r* = 0.71), the expressive language subscale (*r* = 0.72), the sensory awareness subscale (*r* = 0.74), the sociability subscale (*r* = 0.55), and a weaker correlation with the health subscale (*r* = 0.31) [17]. Freire et al. studied 42 autistic children (2–6 years of age) and reported a strong correlation between the CARS score and the total ATEC score (*r* = 0.8), the sociability subscale (*r* > 0.7), the sensory awareness subscale (*r* > 0.7), the expressive language score (*r* > 0.6), and the health score (*r* > 0.6) [32]. Finally, Backer, in a study of 40 children (3–12 years of age), found only a weak correlation between the CARS score and the total ATEC score (*r* = 0.015); no subscale correlations were calculated [33]. The widely ranging correlation reports and low number of participants in the previous studies invite further investigation. This study employed a cross-correlational design, focusing solely on observing the relationships between the parent-reported and clinician-reported measures, without exploring any interventions. The primary goal of this study was to compare the parent-reported MSEC and ATEC scores to the clinician-reported CARS score. The second goal was to investigate the internal consistency of both the MSEC and ATEC assessments in order to improve their acceptance by researchers. A better understanding of the relationship between the MSEC, ATEC, and CARS will improve assessment practices and intervention development for ASD.

## 2. Methods

### 2.1. Participants

Participants were children and adolescents with a diagnosis of ASD who were attending one of the following five locations: (1) Bancroft school in Mt. Laurel, NJ, USA; (2) Somare therapeutic and educational clinic, Foz do Iguaçu, Brazil; (3) Associação Espírita Arco-Íris, Avaré, SP, Brazil; (4) Sociedad Venezolana para Niños y Adultos Autistas, Caracas, Venezuela; or (5) Neato Neuro LLC, Hayden, ID, USA. Participants were recruited by contacting their parents. All children whose parents agreed to participate in the study were included in the study. After obtaining informed consent from a parent, each child was evaluated by a trained clinician using the CARS standard version [31]. In addition, participants’ parents completed the MSEC and ATEC assessments. Neither the clinician nor the parent was aware of the scores generated from their respective completed tests. A statistical analysis was then conducted to examine the relationship between the CARS, on one side, and the MSEC and the ATEC on the other.

A total of 143 participants completed all three assessments. Each child’s ASD diagnosis was confirmed by one of the authors. With 143 participants, the study was powered to detect correlation *r* ≥ 0.23, at the 5% significance level (α = 0.05) and with 80% power (β = 0.2). (Determined using a two-sided test https://www2.ccrb.cuhk.edu.hk/stat/other/correlation.htm, accessed 2 December 2023). The participant age range was from 2 to 22 years (mean 6.7 ± 5.1 years); 81% were males. Data collection and management procedures adhered to stringent ethical standards. All data were collected and stored securely and confidentially. Access to the data was restricted to authorized personnel only, ensuring the privacy and confidentiality of participants.

### 2.2. Childhood Autism Rating Scale (CARS)

Study participants were evaluated using the CARS [31] by clinicians formally trained in the administration of the CARS. The CARS is a 15-item behavioral rating scale developed to quantitatively assess the severity of ASD (Table 2). The CARS works by rating a child’s behavior, characteristics, and abilities against the expected developmental growth of a typical child. Each item is scored from 1 to 4: 1 being normal for a child’s age, 2 being mildly abnormal, 3 being moderately abnormal, and 4 being severely abnormal. The CARS score ranges from 15 (no pathology) to 60 (severe ASD), with 30 being the cutoff rate for a diagnosis of mild autism. Scores 30–37 indicate mild to moderate autism, while scores between 38 and 60 are characterized as severe autism [34].

### 2.3. Autism Treatment Evaluation Checklist (ATEC)

The ATEC questionnaire is composed of four subscales, as follows: (1) speech/language/communication (Table S1), (2) sociability (Table S2), (3) sensory/cognitive awareness (Table S3), and (4) physical/health/behavior (Table S4). The scores from each subscale are combined in order to calculate a total Score, which ranges from 0 to 179 points. A lower score indicates a lower severity of ASD symptoms. The ATEC speech/language/communication subscale primarily measures expressive language and is, therefore, referred to as the expressive language subscale to distinguish it from the complex language comprehension subscale measured using the MSEC evaluation.

### 2.4. Complex Language Comprehension Assessment (MSEC)

The MSEC evaluation (Table S5) was designed to be complementary to the ATEC in measuring complex language comprehension [24,25]. The MSEC consists of 20 questions, the total score ranges from 0 to 40 points; similarly to the ATEC, a lower MSEC score indicates a better developed language comprehension. To simplify the interpretation of figure labels, the MSEC scale is referred to as the complex language comprehension subscale.

### 2.5. Statistical Analysis

Spearman’s correlations were utilized to ascertain the strength of the relationships between the assessments. The choice of Spearman’s correlation was motivated by its robustness to non-normally distributed data and its ability to detect both linear and non-linear relationships between variables.

Additionally, we investigated the factor structure of the MSEC and each of the ATEC’s four subscales using exploratory factor analysis (EFA) and confirmatory factor analysis (CFA). In this case, participant data were randomly split into two equal halves using a random number generator. Half of participants were used for EFA and the other half for CFA.

The ATEC questionnaire is designed to assess four orthogonal facets of ASD using four subscales, as follows: expressive language, sociability, sensory awareness, and health. Accordingly, each subscale was analyzed separately. Analyzing each subscale individually allows for a more clinically meaningful interpretation of the results. It enables the identification of specific areas of strength or difficulty within each subscale. Combining all the ATEC items from the four subscales into a single multidimensional model would have led to an increased complexity of the analysis, which would make it challenging to interpret the results and understand the underlying factor structure, especially when subscales have different scoring ranges and measurement characteristics. While testing a multidimensional model that considers all items together is a valid approach in certain cases, our study aimed to provide a nuanced understanding of the ATEC subscales’ factor structure to gain insights into their unique contributions to ASD assessment. By conducting separate factor analyses, we aimed to explore the latent structure of each subscale and provide clinicians and researchers with more granular information.

Each EFA followed the same methodology, as follows: the minimum residual method was employed to extract the factors and the oblimin method of rotation was used to account for the potential correlations among factors. The number of factors retained was determined using a scree plot and the Kaiser criterion of eigenvalues greater than one. Factor loadings were examined to determine item retention, with a minimum criterion of 0.3 [35]. The model’s convergence was assessed by examining the Root Mean Square of the Residuals (RMSR) and the fit based on off-diagonal values. Sampling adequacy was evaluated using the Kaiser–Meyer–Olkin (KMO) method that measures the proportion of variance among the items that might be common variance. A KMO value > 0.6 is considered adequate for factor analysis, a value above 0.8 is considered good, while a value about 0.9 is considered excellent [36]. Correlations between factor scores and original variables were assessed for convergent validity. The internal consistency of scales was evaluated using Cronbach’s alpha, with acceptable values of alpha ranging from 0.70 to 0.95 [37,38].

Given the non-normal distribution of the data, we employed the Weighted Least Squares Mean and Variance adjusted (WLSMV) estimators in the CFA. The WLSMV was shown to be a robust method for analyzing categorical data and provides accurate parameter estimates and fit indices, even in small sample sizes [39]. CFA was used to test whether the data fit the MSEC and ATEC survey design using the following fit indices: Root Mean Square Error of Approximation (RMSEA, cutoff for good fit < 0.06), Comparative Fit Index (CFI, cutoff for good fit > 0.95), Tucker–Lewis Index (TLI, cutoff for good fit > 0.9), and Standardized Root Mean Square Residual (SRMR, cutoff for good fit < 0.08) [40]. Statistical significance was set at *p* > 0.05. All analyses were conducted using R version 4.1.2.

## 3. Results

**Figure 1:**
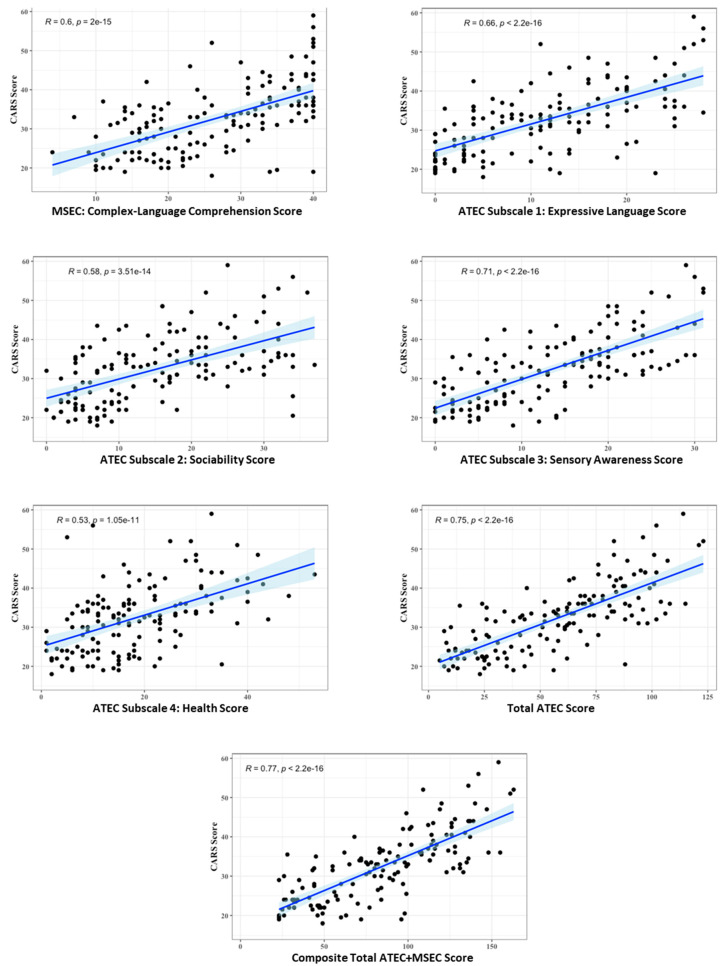
Correlation between the CARS total score and parent-reported assessments.

A total of 143 participants were evaluated by trained clinicians using (1) the Childhood Autism Rating Scale (CARS) [31], as well as by parent-reported (2) assessment of complex language (MSEC) [24], and (3) assessments of expressive language, sociability, sensory awareness, and health (ATEC) [16]. Figure 1 shows correlations between the CARS total score and each parent-reported assessment. The CARS total score correlated strongly with parent reports, as follows: complex language comprehension MSEC (*r* = 0.60, *p* < 0.0001), expressive language (*r* = 0.66, *p* < 0.0001), sociability (*r* = 0.58, *p* < 0.0001), sensory awareness (*r* = 0.71, *p* < 0.0001), health (*r* = 0.53, *p* < 0.0001), and the total ATEC (*r* = 0.75, *p* < 0.0001). The highest correlation was observed between the CARS and the composite score total ATEC + MSEC (*r* = 0.77, *p* < 0.0001).

Table 2 shows Spearman’s correlation between 15 CARS’ items and parent-reported assessments. The strongest correlation was between the CARS’ ‘verbal communication’ item and the expressive language subscale (ATEC subscale 1: *r* = 0.78, *p* < 0.0001). The weakest correlation was between CARS’ ‘fear or nervousness’ item and the health subscale (ATEC subscale 4: *r* = 0.18, *p* < 0.0001). All correlations were statistically significant except one between the CARS’ ‘fear or nervousness’ item and the ATEC 1 expressive language subscale (*r* = 0.14, *p* = 0.1797).

For the complex language comprehension MSEC subscale, the strongest correlation was with the CARS’ ‘verbal communication’ item (*r* = 0.62, *p* < 0.0001) and the weakest correlation was with the CARS’ ‘fear or nervousness’ item (*r* = 0.19, *p* = 0.0418). For the expressive language ATEC 1 subscale, the strongest correlation was with the CARS’ ‘verbal communication’ item (*r* = 0.78, *p* < 0.0001) and the weakest correlation was with the CARS’ ‘fear or nervousness’ item (*r* = 0.14, *p* = 0.1797). For the sociability ATEC 2 subscale, the strongest correlation was with the CARS’ ‘relating to people’ item (*r* = 0.56, *p* < 0.0001) and the weakest correlation was with the CARS’ ‘fear or nervousness’ item (*r* = 0.20, *p* = 0.0197). For the sensory awareness ATEC 3 subscale, the strongest correlation was with the CARS’ ‘verbal communication’ item (*r* = 0.69, *p* < 0.0001) and the weakest correlation was with the CARS’ ’fear or nervousness’ item (*r* = 0.23, *p* = 0.0028). For the health ATEC 4 subscale, the strongest correlation was with the CARS’ ’verbal communication’ item (*r* = 0.47, *p* < 0.0001) and the weakest correlation was with the CARS’ ’fear or nervousness’ item (*r* = 0.18, *p* = 0.0142).

For the ATEC total, the strongest correlation was with the CARS’ ‘verbal communication’ item (*r* = 0.71, *p* < 0.0001) and the weakest correlation was with the CARS’ ‘fear or nervousness’ item (*r* = 0.25, *p* = 0.0043). For the ATEC total + MSEC, the strongest correlation was with the CARS’ ‘verbal communication’ item (*r* = 0.74, *p* < 0.0001) and the weakest correlation was with the CARS’ ‘fear or nervousness item’ (*r* = 0.25, *p* = 0.0042).

### 3.1. Internal Consistency

Internal consistency was evaluated using Cronbach’s alpha. For all subscales, Cronbach’s alpha exceeded 0.7, which indicated a good internal consistency, as follows: the MSEC complex language comprehension subscale α = 0.96; ATEC 1 expressive language subscale α = 0.95; ATEC 2 sociability subscale α = 0.92; ATEC 3 sensory awareness subscale α = 0.92; ATEC 4 health subscale α = 0.88; ATEC total α = 0.93; composite ATEC total + MSEC α = 0.96; CARS α = 0.93.

### 3.2. Exploratory Factor Analysis of the Complex Language Assessment MSEC

Participants were randomly split into two equal halves using a random number generator. Half of participants were used here for exploratory factor analysis (EFA) and the other half were used later for confirmatory factor analysis (CFA). EFA was performed using the Spearman’s correlation method with the minimum residual estimation technique and oblimin rotation. The Kaiser–Meyer–Olkin (KMO) value of 0.90 indicated that the items were suitable for factor analysis, Table S6. The results of the EFA support the notion of the MSEC’s unidimensionality, with a single factor explaining 71% of the total variance. The standardized factor loadings for the items ranged from 0.51 to 0.94, with 18 out of the 20 items exceeding 0.70. For the two items with lowest loadings (item 18 and item 19), item communalities were also smaller (0.43 and 0.26, respectively). All other item communalities ranged from 0.52 to 0.87, indicating that the single factor accounted for a substantial proportion of the variance in each item. The Root Mean Square of the Residuals (RMSR) was 0.16, with a degree-freedom-corrected RMSR of 0.17, demonstrating an acceptable model fit. Furthermore, the off-diagonal fit value of 0.95 suggests an adequate single-factor model fit for the MSEC assessment. In addition to the exploratory factor analysis, correlations between the factor scores and the original variables were examined. The correlations were lowest for MSEC item 19 (*r* = 0.44) and highest for MSEC item 13 (*r* = 0.80).

### 3.3. Confirmatory Factor Analysis of the Complex Language Assessment MSEC

In addition to the EFA, a confirmatory factor analysis (CFA) was conducted to further examine the unidimensional factor structure of the MSEC. The CFA was performed using the Weighted Least Squares Mean and Variance adjusted estimator and polychoric correlations to account for the ordinal nature of the data. The CFA model specified a single factor with all 20 items as indicators and provided evidence for the adequacy of the proposed single-factor model. The Comparative Fit Index (CFI) was 1.00, and the Tucker–Lewis Index (TLI) was 0.99, indicating a good model fit, as values greater than 0.95 are generally considered acceptable, Table S7. The Root Mean Square Error of Approximation (RMSEA) was 0.075, and the Standardized Root Mean Square Residual (SRMR) was 0.124. Although the RMSEA value is slightly above the 0.05 threshold for a good fit and the SRMR is higher than the desired threshold of 0.08, the overall fit indices suggest that the single-factor model is a reasonable representation of the data. The standardized factor loadings for the 20 items ranged from 0.30 (item 19) to 0.88 (item 20), indicating that all items were significantly related to the underlying complex language comprehension factor (*p* < 0.01). The remaining factor loadings showed moderate to high associations with the latent construct. Overall, the CFA results support the unidimensionality of the MSEC assessment and provide further evidence for its construct validity.

### 3.4. Exploratory Factor Analysis of the ATEC Subscales

An exploratory factor analysis (EFA) was implemented separately on each of the four subscales of the Autism Treatment Evaluation Checklist (ATEC), using the minimum residual method coupled with oblimin rotation. The sampling adequacy evaluated using the KMO statistic indicated that factor analysis for all but one subscale (ATEC 4 health subscale, KMO = 0.68) is suitable for these subscales.

The ATEC 1 expressive language subscale demonstrated the highest proportion of variance (78%) encapsulated within a one-factor model, bearing standardized item loadings spanning from 0.72 to 0.99, Table S8. The model fit was satisfactory, with a Root Mean Square Residual (RMSR) of 0.15 and an off-diagonal fit value of 0.97.

For the ATEC 2 sociability subscale, the one-factor model accounted for 60% of the variance, Table S9. All items, excluding items 14 and 15, exhibited standardized loadings exceeding 0.58, indicating a robust association with the underlying factor. Items 14 and 15 had standardized item loadings of 0.37 and 0.30, respectively, contributing minimally to the item variance explained by the one-factor model (0.14 and 0.09, respectively). Despite an acceptable RMSR and off-diagonal fit of 0.18 and 0.91, respectively, excluding items with relatively low factor loading augmented the variance explained by the model to 67%, Table S10. Additionally, the RMSR decreased marginally to 0.16 and the off-diagonal fit value improved to 0.95, signifying a superior model fit post item removal.

The one-factor model for the ATEC 3 sensory awareness subscale accounted for 68% of the variance, with high standardized loadings for all items, ranging from 0.63 to 0.97, Table S11. Although the model fit was superior to that of Subscale 2, it fell short of Subscale 1, as evidenced by an RMSR of 0.16 and an off-diagonal fit value of 0.95.

The ATEC 4 health subscale had the lowest proportion of variance explained by a one-factor model (38%) and exhibited the broadest range of standardized loadings, from 0.17 to 0.91, Table S12. The health subscale manifested the poorest fit among the four subscales, indicated by an RMSR of 0.22 and an off-diagonal fit value of 0.76. Removing items with standardized loadings of less than 0.55 (items 4, 5, 10, 15, 16, and 17) increased the proportion of variance explained by the one-factor model to 52%, Table S13.

### 3.5. Confirmatory Factor Analysis of the ATEC Subscales

For each ATEC subscale, a confirmatory factor analysis (CFA) was also performed. CFA parameters were estimated using a diagonal weighted least squares estimator. For the ATEC 1 expressive language subscale, the CFA model displayed an excellent fit, based on the Comparative Fit Indices (CFI = 0.99) and Tucker–Lewis Indices (TLI = 0.99), surpassing the recommended threshold of 0.95, Table S14. The Root Mean Square Error of Approximation demonstrated a good fit (RMSEA = 0.05). SRMR, at 0.12, exceeded the recommended threshold of 0.08, hinting at potential model improvement. All items had significant loadings onto the latent factor (*p* < 0.01). The latent factor variance itself was not significant, indicating minor variation across the sample.

For the ATEC 2 sociability subscale, the CFA model showed a good fit based on the CFI (0.87) and TLI (0.86), surpassing the typical threshold, Table S15. Similarly, the RMSEA (0.08) indicated a good fit. All observed items showed statistically significant factor loadings onto the latent variable (*p* < 0.0001), except for items 14 (*p* = 0.1510) and 15 (*p* = 0.4220). Removing these items led to an improvement in both the CFI (0.93) and the TLI (0.92). The resulting RMSEA (0.06) and SRMR (0.8) indicated a better model fit, Table S16.

For the ATEC 3 sensory awareness subscale, the CFA model indicated a good fit based on the CFI (0.98) and TLI (0.98). The model had an RMSEA of 0.05 and an SRMR of 0.11, Table S17. All items in the sensory awareness subscale significantly loaded onto the latent factor (*p* < 0.05), except for item 4 (*p* = 0.0750). The one-factor structure suggested by these loadings, coupled with residual variances between 0.12 and 0.65, supports the model’s good fit to the data, despite the non-significant variance of the latent variable (*p* = 0.172).

For the ATEC 4 health subscale, the CFA model suggested a moderate fit, Table S18. The CFI (0.89) and TLI (0.88) values fell short of the ideal threshold of >0.95, indicating a satisfactory but not exceptional fit. The RMSEA value of 0.06, considered moderate, further corroborated this interpretation. However, the SRMR value of 0.13 exceeded the widely accepted cutoff of 0.08, indicating a potential model misfit in terms of the residuals. Removing items 4, 5, 10, 15, 16, and 17 improved CFI (0.92) and TLI (0.91), which surpassed the thresholds of 0.95 and 0.90, respectively, suggesting a model improvement, Table S19. However, the RMSEA (0.07) was still found to be slightly higher than the cutoff of 0.06 and the SRMR (0.13) was found to be higher than the cutoff of 0.08, indicating a moderate fit to the data. This is also shown by the item residuals, with all the unique variances being statistically significant (*p* < 0.05).

## 4. Discussion

This is the largest cross-sectional correlational study comparing the parent-reported ATEC to the clinician-scored CARS. This is also the first study comparing the complex language comprehension parent-reported MSEC to the CARS. Five clinics across the world were involved in the study with a combined number of 143 participants, from 2 to 22 years of age (mean 6.7 ± 5.1). The CARS score correlated strongly with ATEC subscale 1 expressive language (*r* = 0.66, *p* < 0.0001), ATEC subscale 2 sociability (*r* = 0.58, *p* < 0.0001), ATEC subscale 3 sensory awareness (*r* = 0.71, *p* < 0.0001), and ATEC subscale 4 health (*r* = 0.53, *p* < 0.0001), as well as with the total ATEC score (*r* = 0.75, *p* < 0.0001), Figure 1. The strong correlation between the ATEC and CARS is similar to that reported by other investigators [17,32] and suggests a reasonable fidelity of the ATEC assessment (Table 3). While Backer reached an opposite conclusion and reported correlation between the CARS and ATEC of *r* = 0.015, *p* = 0.926 [33], his study had multiple irregularities (e.g., a small number of participants and did not include reporting a correlation between the CARS and individual ATEC subscales).

Although larger than prior studies, a sample size of 143 participants remains relatively modest. Future studies should compare parent-reported and clinician-reported measures in a greater number of participants.

The complex language comprehension subscale MSEC adds an important orthogonal axis to the ATEC subscales. The complex language comprehension score alone was found to be 90% correct at predicting ASD teenagers’ high-functioning versus low-functioning class assignment, compared to the only 50% accuracy manifested using the IQ score [23]. Furthermore, the MSEC but none of the ATEC subscales demonstrated the benefit of the gluten-free diet and raw-vegetable-eating [30], as well as the detriment of high video and TV use [27]. Furthermore, the MSEC was more sensitive than the ATEC subscales to the negative effect of sleep problems [41] and seizures [42], as well as to the positive effects of early language intervention [28], joint engagement with an adult [43], pretend play [29], as well as meat-, egg-, and vegetable-eating [30].

This study’s results demonstrate that the MSEC is strongly correlated to the CARS (*r* = 0.60, *p* < 0.0001), despite the CARS’ items not directly assessing complex language comprehension, Table 2. Our results show the strongest correlation between the MSEC and the CARS’ item 11 ‘verbal communication’ (*r* = 0.62, *p* < 0.0001) and item 2 ‘imitation’ (*r* = 0.59, *p* < 0.0001). All the other CARS’s items correlations to the MSEC were less than 0.55; the weakest correlation was observed between the MSEC and the CARS item 10 ‘fear or nervousness’ (*r* = 0.19, *p* < 0.05).

The composite of all five parent-reported scores demonstrated a stronger correlation to the CARS (total ATEC + MSEC, *r* = 0.77, *p* < 0.0001) than the total ATEC score alone (*r* = 0.75, *p* < 0.0001). These results suggest a high fidelity of the MSEC and ATEC parent reports and, especially, of their composite score, total ATEC + MSEC. In order to better understand the MSEC and ATEC subscales, we investigated their internal consistency and conducted factor analysis.

### 4.1. MSEC Complex Language Comprehension analysis of Unidimensionality

The MSEC complex language comprehension subscale Cronbach’s alpha was 0.96, which indicates an excellent internal consistency. The results of the exploratory factor analysis (EFA) and the confirmatory factor analysis (CFA) strongly support the notion of the MSEC’s unidimensionality. A single factor explained 71% of the total variance. The Root Mean Square of the Residuals (RMSR) was 0.16, with a degree-of-freedom corrected RMSR of 0.17, demonstrating an acceptable model fit. Furthermore, the off-diagonal fit value of 0.95 suggests an adequate single-factor model fit for the MSEC assessment. The Comparative Fit Index (CFI) was 0.998, and the Tucker–Lewis Index (TLI) was 0.986, indicating a good model fit. The Root Mean Square Error of Approximation (RMSEA) was 0.075 and the Standardized Root Mean Square Residual (SRMR) was 0.124. Although the RMSEA value is slightly above the 0.05 threshold for a good fit and the SRMR is higher than the desired threshold of 0.08, the overall fit indices suggest that the single-factor model is a reasonable representation of the data. All items had significant loadings onto the latent factor (*p* < 0.01). These results indicate that all the MSEC items are significantly related to the underlying complex language comprehension factor. 

### 4.2. ATEC 1 Expressive Language Subscale Analysis of Unidimensionality

The ATEC 1 expressive language subscale Cronbach’s alpha was 0.95, which indicates an excellent internal consistency. The results of the EFA and CFA strongly support the notion of the ATEC’s subscale 1 unidimensionality. A single factor explained 78% of the total variance. The RMSR was 0.15, demonstrating an acceptable model fit. Furthermore, the off-diagonal fit value of 0.97 suggests an adequate single-factor model fit for this subscale. The CFI was 0.994 and the TLI was 0.986, surpassing the recommended threshold of 0.95. The RMSEA (=0.048) demonstrated a good fit. The SRMR (=0.118) exceeded the recommended threshold of 0.08, hinting at a potential model improvement. All items had significant loadings onto the latent factor (*p* < 0.01). The latent factor variance itself was not significant, indicating a minor variation across the sample. These results indicate that all items are significantly related to the underlying expressive language factor.

### 4.3. ATEC 2 Sociability Subscale Analysis of Unidimensionality

The ATEC 2 sociability subscale Cronbach’s alpha was 0.92, which indicates a good internal consistency. A single factor explained 60% of the total variance. The RMSR was 0.18, demonstrating an acceptable model fit. Furthermore, the off-diagonal fit value of 0.91 suggests an adequate single-factor model fit for this subscale. The CFI was 0.873 and the TLI was 0.858, just below the recommended threshold of 0.95. The RMSEA (=0.075) demonstrated a good fit. The SRMR (=0.094) was below the recommended threshold of 0.08. All items showed statistically significant factor loadings onto the latent variable (*p* < 0.0001) except item 14 (“Disagreeable/not compliant”, *p* = 0.151) and item 15 (“Temper tantrums”, *p* = 0.422). These results indicate that 18 out of 20 items are significantly related to the underlying sociability factor.

### 4.4. ATEC 3 Sensory Awareness Subscale Analysis of Unidimensionality

The ATEC 3 sensory awareness subscale Cronbach’s alpha was 0.92, which indicates an excellent internal consistency. The results of the EFA and CFA strongly support the notion of the ATEC’s subscale 3 unidimensionality. A single factor explained 68% of the total variance. The RMSR was 0.16, demonstrating an acceptable model fit. Furthermore, the off-diagonal fit value of 0.95 suggests an adequate single-factor model fit for this subscale. The CFI was 0.980 and the TLI was 0.977, surpassing the recommended threshold of 0.95. The RMSEA (=0.053) demonstrated a good fit. The SRMR (=0.110) exceeded the recommended threshold of 0.08, hinting at a potential model improvement. All items had significant loadings onto the latent factor (*p* < 0.05) except for item 4 (“Looks at pictures and T.V.”, *p* = 0.075). The latent factor variance itself was not significant, indicating a minor variation across the sample. These results indicate that 17 out of 18 items are significantly related to the underlying sensory awareness factor.

### 4.5. ATEC 4 Health Subscale Analysis of Unidimensionality

The ATEC 4 health subscale contains a list of 25 various health problems that are unrelated or poorly related. Accordingly, we did not expect to see unidimensionality in this subscale. As expected, the ATEC 4 health subscale had the lowest Cronbach’s alpha of 0.88 and had the least proportion of variance explained by a one-factor model (38%). The RMSR of 0.22 and an off-diagonal fit value of 0.76 suggest a poor model fit. The CFI (0.887) and TLI (0.876) values fell short of the ideal threshold of >0.95, indicating a satisfactory but not exceptional fit. The RMSEA value of 0.060, considered moderate, further confirmed this interpretation. These results indicate that items of the health subscale are poorly related to a single factor.

### 4.6. Clinical Implications

The development and validation of novel treatments for children with ASD can be facilitated by studies in which parents are trained to administer a treatment [10,13,15,44]. In order to succeed, such studies must be longitudinal to ensure that the treatment effect can be reliably measured, since small improvements enabled by an early treatment may not result in a significant score change when tested in a few months; however, once amplified over the years, the effect can be often consistently assessed when measured after a period of several years [4]. The use of clinicians to assess children over many years contributes significantly to the cost of a study. To keep costs manageable, longitudinal studies involving a large number of participants can use parent-reported assessments. Although parents may succumb to wishful thinking and inflate their child’s abilities on a single assessment [45], the trend of changes observed through the measurement of score dynamics across multiple assessments spanning several years, generates a meaningful outcome measure, especially when parents are blinded to their treatment versus control group allocation. Furthermore, parents possess a deep understanding of their children. This understanding may be especially important for complex language comprehension assessments, which are tricky to evaluate in a clinical setting. Numerous prior studies indicate that parent reports align closely with direct clinician assessments [46,47], and analyses from our database further corroborate the reliability and accuracy of parent reports [26,27,41]. Not surprisingly, longitudinal studies often employ parent-reported assessments of child development as an outcome measure [12,48,49,50,51]. This study adds confidence to the parent-reported MSEC and ATEC assessments.

Overall, this study provides evidence that parent-reported outcome measures can be a valuable tool for assessing the effects of interventions for children with ASD. However, it is important to note that, when feasible, these measures should be complemented by clinician-administered measures and other objective measures to provide a comprehensive picture of a child’s development.

## Figures and Tables

**Table 1:** The complex language comprehension scale (measured using the MSEC) is more sensitive to children development than the expressive language scale.

	Study	Complex Language Comprehension	Expressive Language	Sociability	Sensory Awareness	Health
1	High-TV users—Low-TV users (N = 3227) [27]	**2.58 (1.04; 0.0128)**	−1.26 (0.7; 0.0719)	1.82 (0.99; 0.0663)	1.58 (0.85; 0.0631)	1.05 (1.52; 0.4898)
2	Control—Language therapy (MITA) (N = 6454) [28]	**4.74 (1.13; <0.0001)**	**1.84 (0.77; 0.0172)**	0.46 (1.05; 0.6584)	0.24 (0.9; 0.7886)	2.57 (1.62; 0.1119)
3	No-pretend-play—Pretend-play (N = 7069) [29]	**7.26 (0.72; <0.0001)**	**2.95 (0.53; <0.0001)**	0.24 (0.72; 0.7348)	**1.99 (0.6; 0.0009)**	−0.76 (1.08; 0.4782)
4	Control —Gluten-free diet (N = 5553) [30]	**2.97 (0.75; <0.0001)**	0.3 (0.56; 0.5918)	−0.02 (0.76; 0.9746)	0.93 (0.64; 0.1513)	−0.1 (1.17; 0.9339)
5	Control—Meat-eating (N = 5553) [30]	**2.4 (0.56; <0.0001)**	**0.92 (0.42; 0.0279)**	0.93 (0.55; 0.0944)	**1.89 (0.46; <0.0001)**	0.78 (0.83; 0.3467)
6	Control—Vegetable-eating (N = 5553) [30]	**2.49 (0.53; <0.0001)**	**0.99 (0.4; 0.0137)**	0.6 (0.53; 0.2537)	**1.74 (0.45; 0.0001)**	0.73 (0.79; 0.3538)
7	Control—Raw-vegetable-eating (N = 5553) [30]	**3.11 (0.74; <0.0001)**	0.66 (0.55; 0.2307)	0.9 (0.73; 0.2165)	**1.97 (0.62; 0.0014)**	**−2.46 (1.08; 0.0222)**

The table shows score differences between groups at the end of a three-year study period (Month 36). For every study, children in both groups were matched by age, gender, complex language comprehension, expressive language, sociability, sensory awareness, and health at the first evaluation (baseline) using propensity score analysis. Children’s age at baseline in all studies was 2 to 5 years. The differences between groups are presented as LS Mean (SE; *p*-value). A positive difference indicates a greater final score (more severe symptoms) in the first-listed group compared to the second-listed group. Bold values denote statistical significance at the *p* < 0.05 level.

**Table 2:** Spearman’s correlation between the CARS items and parent-reported subscales. Correlations ≥ 0.6 were bolded. All *p*-values < 0.0001 unless marked: ** < 0.01; * < 0.05; ^ >0.05.

	Complex Language Comprehension	Expressive Language	Sociability	Sensory Awareness	Health	ATEC Total	ATEC Total + MSEC
**CARS Total**	**0.6**	**0.66**	0.58	**0.71**	0.53	**0.75**	**0.77**
**1. Relating to People**	0.44	0.53	0.56	**0.65**	0.39	**0.65**	**0.65**
**2. Imitation**	0.59	**0.64**	0.49	**0.63**	0.35	**0.63**	**0.67**
**3. Emotional Response**	0.35	0.38	0.40	0.47	0.46	0.52	0.52
**4. Body Use**	0.37	0.48	0.23 **	0.44	0.40	0.46	0.47
**5. Object Use**	0.55	0.59	0.51	**0.63**	0.45	**0.66**	**0.68**
**6. Adaption to Change**	0.20 *	0.24 *	0.36	0.32	0.46	0.43	0.40
**7. Visual Response**	0.48	0.56	0.49	**0.60**	0.33	**0.6**	**0.61**
**8. Listening Response**	0.36	0.42	0.40	0.47	0.25 **	0.46	0.47
**9. Taste, Smell, Touch**	0.36	0.37	0.37	0.45	0.44	0.51	0.50
**10. Fear or Nervousness**	0.19 *	0.14 ^	0.20 *	0.23 **	0.18 *	0.25 **	0.25 **
**11. Verbal communication**	**0.62**	**0.78**	0.51	**0.69**	0.47	**0.71**	**0.74**
**12. Nonverbal communication**	0.48	0.53	0.48	0.54	0.38	0.59	**0.60**
**13. Activity Level**	0.36	0.32	0.35	0.39	0.27	0.41	0.43
**14. Intellectual Response**	0.45	0.44	0.48	0.58	0.30	0.54	0.55
**15. General Impression**	0.51	0.57	0.37	0.49	0.40	0.56	0.59

**Table 3:** Summary of studies that investigated the correlation between the CARS score and the MSEC/ATEC subscale scores.

Study	Age Range (Years)	MSEC Complex Language Comprehension	ATEC 1: Expressive Language	ATEC 2: Sociability	ATEC 3: Sensory Awareness	ATEC 4: Health	ATEC Total
This study (N = 143)	2–22	0.6	0.66	0.58	0.71	0.53	0.75
Geier et al. (N = 56) [17]	2–16	no data	0.72	0.55	0.74	0.31	0.71
Freire et al. (N = 42) [32]	2–6	no data	>0.6	>0.7	>0.7	>0.6	0.8
Backer (N = 40) [33]	3–12	no data	no data	no data	no data	no data	0.015

## Data Availability

The unidentified raw data from this manuscript are available from the corresponding author upon reasonable request.

## Supplementary Materials

The following supporting information can be downloaded at: https://www.mdpi.com/article/10.3390/pediatric16010016/s1. Table S1. ATEC subscale 1: Speech/Language/Communication. The answers choices were: very true (0 points), somewhat true (1 point), and not true (2 points). The subscale score ranges from 0 to 28, Table S2. ATEC subscale 2: Sociability. The answers choices were: very true (0 points), somewhat true (1 point), and not true (2 points). The subscale score ranges from 0 to 40, Table S3. ATEC subscale 3: Sensory/Cognitive awareness. The answers choices were: very true (0 points), somewhat true (1 point), and not true (2 points). The subscale score ranges from 0 to 36, Table S4. ATEC subscale 4: Health/Physical/Behavior. The answers choices were: not a problem (0 points), minor problem (1 point), moderate problem (2 points), and serious problem (3 points). The subscale score ranges from 0 to 75, Table S5. MSEC prepositional language comprehension subscale. The answers choices were: very true (0 points), somewhat true (1 point), and not true (2 points), Table S6: Exploratory Factor Analysis of MSEC Complex-language comprehension, Table S7. Confirmatory Factor Analysis of MSEC Complex-language comprehension, Table S8. Exploratory Factor Analysis of ATEC Subscale 1 Expressive Language, Table S9. Exploratory Factor Analysis of ATEC Subscale 2 Sociability, Table S10. Exploratory Factor Analysis of ATEC Subscale 2 Sociability after removing items 14 and 15, Table S11: Exploratory Factor Analysis of ATEC Subscale 3 Cognitive Awareness, Table S12. Exploratory Factor Analysis of ATEC Subscale 4 Health, Table S13. Exploratory Factor Analysis of ATEC Subscale 4 Health after removing items 4, 5, 10, 15, 16, and 17, Table S14: Confirmatory Factor Analysis of ATEC Subscale 1 Expressive Language, Table S15. Confirmatory Factor Analysis of ATEC Subscale 2 Sociability, Table S16. Confirmatory Factor Analysis of ATEC Subscale 2 Sociability after removing items 14 and 15, Table S17. Confirmatory Factor Analysis of ATEC Subscale 3 Sensory Awareness, Table S18: Confirmatory Factor Analysis of ATEC Subscale 4 Health, Table S19. Confirmatory Factor Analysis of ATEC Subscale 4 Health after removing items 4, 5, 10, 15, 16, and 17.
