# Supplementary material for: A Comparison of Parent Reports, the Mental Synthesis Evaluation Checklist (MSEC) and the Autism Treatment Evaluation Checklist (ATEC), with the Childhood Autism Rating Scale (CARS)
Source: Pediatr Rep. 2024 Mar 11;16(1):174–89. doi: 10.3390/pediatric16010016 (PMC10975750; doi:10.3390/pediatric16010016)
Supplement: Supplementary file 1 [file pediatrrep-16-00016-s001.zip › pediatrrep-2782591-supplementary.pdf]

<u>Supplementary Material</u>

Table S1. ATEC subscale 1: Speech/Language/Communication. The answer choices were very true (0 points), somewhat true (1 point), and not true (2 points).

1. Knows own name
2. Responds to ‘No’ or ‘Stop’
3. Can follow some commands
4. Can use one word at a time (No, Eat, Water, etc.)
5. Can use two words at a time (Don't want, Go home)
6. Can use three words at a time (Want more milk)
7. Knows 10 or more words
8. Can use sentences with four or more words
9. Explains what he/she wants
10. Asks meaningful questions
11. Speech tends to be meaningful/relevant
12. Often uses several successive sentences
13. Carries on fairly good conversation
14. Has normal ability to communicate for his/her age

Table S2. ATEC subscale 2: Sociability. The answer choices were very true (0 points), somewhat true (1 point), and not true (2 points).

1. Seems to be in a shell – you cannot reach him/her
2. Ignores other people
3. Pays little or no attention when addressed
4. Uncooperative and resistant
5. No eye contact
6. Prefers to be left alone
7. Shows no affection
8. Fails to greet parents
9. Avoids contact with others
10. Does not imitate
11. Dislikes being held/cuddled
12. Does not share or show
13. Does not wave ‘bye bye’
14. Disagreeable/not compliant
15. Temper tantrums
16. Lacks friends/companions
17. Rarely smiles
18. Insensitive to other's feelings
19. Indifferent to being liked
20. Indifferent if parent(s) leave

Table S3. ATEC subscale 3: Sensory/Cognitive awareness. The answer choices were very true (0 points), somewhat true (1 point), and not true (2 points).

1. Responds to own name
2. Responds to praise
3. Looks at people and animals
4. Looks at pictures (and T.V.)
5. Does drawing, coloring, art
6. Plays with toys appropriately
7. Appropriate facial expression
8. Understands stories on T.V.
9. Understands explanations
10. Aware of environment
11. Aware of danger
12. Shows imagination
13. Initiates activities
14. Dresses self
15. Curious, interested
16. Venturesome—explores
17. “Tuned in”—Not spacey
18. Looks where others are looking

Table S4. ATEC subscale 4: Health/Physical/Behavior. The answer choices were not a problem (0 points), minor problem (1 point), moderate problem (2 points), and serious problem (3 points).

1. Bed-wetting
2. Wets pants/diapers
3. Soils pants/diapers
4. Diarrhea
5. Constipation
6. Sleep problems
7. Eats too much/too little
8. Extremely limited diet
9. Hyperactive
10. Lethargic
11. Hits or injures self
12. Hits or injures others
13. Destructive
14. Sound-sensitive
15. Anxious/fearful
16. Unhappy/crying
17. Seizures
18. Obsessive speech
19. Rigid routines
20. Shouts or screams
21. Demands sameness
22. Often agitated
23. Not sensitive to pain
24. “Hooked” or fixated on certain objects/topics
25. Repetitive movements (stimming, rocking, etc.)

Table S5. MSEC prepositional language comprehension subscale. The answer choices were very true (0 points), somewhat true (1 point), and not true (2 points).

1. Understands simple stories that are read aloud
2. Understands elaborate fairy tales that are read aloud (i.e. stories describing FANTASY creatures)
3. Draws a VARIETY of RECOGNIZABLE images (objects, people, animals, etc.)
4. Can draw a NOVEL image following YOUR description (e.g. a three-headed horse)
5. Engages in a VARIETY of make-believe activities (such as: playing house, playing with toy soldiers, building forts and castles, etc.)
6. Understands some simple modifiers (i.e. green apple vs. red apple or big apple vs. small apple)
7. Understands several modifiers in a sentence (i.e. small green apple)
8. Understands size (can select the largest/smallest object out of a collection of objects)
9. Understands possessive pronouns (i.e. your apple vs. her apple)
10. Understands spatial prepositions (i.e. put the apple ON TOP of the box vs. INSIDE the box vs. BEHIND the box)
11. Understands verb tenses (i.e. I will eat an apple vs. I ate an apple)
12. Understands the change in meaning when the order of words is changed (i.e. understands the difference between 'a cat ate a mouse' vs. 'a mouse ate a cat')
13. Understands NUMBERS (i.e. two apples vs. three apples)
14. Can perform simple arithmetic: 2 + 3 = ?
15. Can add larger numbers: 7 + 6 = ?
16. Can perform simple subtraction: 3 – 2 = ?
17. Can subtract larger numbers: 15 – 7 = ?
18. Can perform simple multiplication: 2 × 2 = ?
19. Can multiply larger numbers: 6 × 7 = ?
20. Understands explanations about people, objects, or situations beyond their immediate surroundings (e.g., “Mom is walking the dog,” “The snow has turned to water”)

Table S6: Exploratory Factor Analysis of MSEC complex language comprehension

Items	Factor Loadings	Factor Score Correlations	Communality	Measure of Sampling Adequacy
X1.MSEC	0.87	0.74	0.75	0.87
X2.MSEC	0.85	0.73	0.73	0.85
X3.MSEC	0.90	0.77	0.82	0.93
X4.MSEC	0.86	0.73	0.73	0.92
X5.MSEC	0.85	0.73	0.72	0.93
X6.MSEC	0.82	0.70	0.68	0.90
X7.MSEC	0.89	0.76	0.79	0.93
X8.MSEC	0.88	0.75	0.77	0.92
X9.MSEC	0.93	0.79	0.86	0.95
X10.MSEC	0.92	0.78	0.84	0.95
X11.MSEC	0.91	0.78	0.84	0.88
X12.MSEC	0.90	0.77	0.80	0.88
X13.MSEC	0.94	0.80	0.87	0.96
X14.MSEC	0.82	0.70	0.68	0.83
X15.MSEC	0.77	0.66	0.59	0.86
X16.MSEC	0.80	0.68	0.64	0.90
X17.MSEC	0.72	0.62	0.52	0.91
X18.MSEC	0.66	0.56	0.43	0.87
X19.MSEC	0.51	0.44	0.26	0.84
X20.MSEC	0.91	0.77	0.82	0.95

Kaiser–Meyer–Olkin Value = 0.9
Fit Based Upon Off-Diagonal Values = 0.95
Root Mean Square of the Residuals = 0.16
Sum of the Squared Loadings = 14.15
Proportion Variance = 71%
Cronbach's Alpha = 0.96 (95% CI: 0.84, 0.97)

Table S7: Confirmatory Factor Analysis of MSEC complex language comprehension

Items	Standardized Factor Loadings	P - Value
X1.MSEC	0.73	<0.0001
X2.MSEC	0.84	<0.0001
X3.MSEC	0.66	<0.0001
X4.MSEC	0.75	<0.0001
X5.MSEC	0.76	<0.0001
X6.MSEC	0.70	<0.0001
X7.MSEC	0.82	<0.0001
X8.MSEC	0.77	<0.0001
X9.MSEC	0.81	<0.0001
X10.MSEC	0.81	<0.0001
X11.MSEC	0.85	<0.0001
X12.MSEC	0.83	<0.0001
X13.MSEC	0.74	<0.0001
X14.MSEC	0.72	<0.0001
X15.MSEC	0.65	<0.0001
X16.MSEC	0.72	<0.0001
X17.MSEC	0.62	<0.0001
X18.MSEC	0.47	<0.0001
X19.MSEC	0.30	0.008
X20.MSEC	0.88	<0.0001

Root Mean Square Error of Approximation = 0.075 (90% CI: 0.059, 0.090)
Comparative Ft Index = 0.998
Tucker–Lewis Index = 0.986
Standardized Root Mean Square Residual = 0.124

Table S8: Exploratory Factor Analysis of ATEC Subscale 1 Expressive Language

Items	Factor Loadings	Factor Score Correlations	Communality	Measure of Sampling Adequacy
X1.ATEC1	0.72	0.61	0.52	0.88
X2.ATEC1	0.79	0.67	0.62	0.84
X3.ATEC1	0.84	0.71	0.70	0.88
X4.ATEC1	0.78	0.66	0.61	0.88
X5.ATEC1	0.86	0.73	0.74	0.87
X6.ATEC1	0.92	0.78	0.85	0.86
X7.ATEC1	0.94	0.79	0.88	0.95
X8.ATEC1	0.93	0.79	0.87	0.91
X9.ATEC1	0.99	0.84	0.99	0.92
X10.ATEC1	0.95	0.80	0.90	0.95
X11.ATEC1	0.97	0.82	0.94	0.96
X12.ATEC1	0.89	0.75	0.79	0.92
X13.ATEC1	0.91	0.77	0.83	0.92
X14.ATEC1	0.84	0.72	0.71	0.92

Kaiser–Meyer–Olkin Value = 0.91
Fit Based Upon Off-Diagonal Values = 0.97
Root Mean Square of the Residuals = 0.15
Sum of the Squared Loadings = 10.94
Proportion Variance = 78%
Cronbach's Alpha = 0.95 (95% CI: 0.93, 0.96)

Table S9: Exploratory Factor Analysis of ATEC Subscale 2 Sociability

**Items**	**Factor Loadings**	**Factor Score Correlations**	**Communality**	**Measure of Sampling Adequacy**
X1.ATEC2	0.74	0.59	0.54	0.86
X2.ATEC2	0.86	0.68	0.74	0.91
X3.ATEC2	0.78	0.62	0.61	0.86
X4.ATEC2	0.58	0.46	0.34	0.78
X5.ATEC2	0.75	0.59	0.56	0.84
X6.ATEC2	0.82	0.65	0.68	0.84
X7.ATEC2	0.88	0.70	0.77	0.82
X8.ATEC2	0.81	0.64	0.65	0.83
X9.ATEC2	0.76	0.60	0.58	0.80
X10.ATEC2	0.77	0.61	0.59	0.86
X11.ATEC2	0.87	0.69	0.76	0.87
X12.ATEC2	0.84	0.67	0.70	0.85
X13.ATEC2	0.82	0.65	0.67	0.77
**X14.ATEC2**	**0.37**	**0.30**	**0.14**	**0.71**
**X15.ATEC2**	**0.30**	**0.24**	**0.09**	**0.59**
X16.ATEC2	0.89	0.71	0.79	0.89
X17.ATEC2	0.86	0.68	0.73	0.90
X18.ATEC2	0.89	0.71	0.79	0.88
X19.ATEC2	0.84	0.67	0.71	0.89
X20.ATEC2	0.79	0.63	0.62	0.84

Kaiser–Meyer–Olkin Value = 0.84
Fit Based Upon Off-Diagonal Values = 0.91
Root Mean Square of the Residuals = 0.18
Sum of the Squared Loadings = 12.06
Proportion Variance = 60%
Cronbach's Alpha = 0.92 (95% CI: 0.89, 0.94)

Table S10: Exploratory Factor Analysis of ATEC Subscale 2 Sociability after removing items 14 and 15

Items	Factor Loadings	Factor Score Correlations	Communality	Measure of Sampling Adequacy
X1.ATEC2	0.72	0.57	0.52	0.85
X2.ATEC2	0.87	0.69	0.76	0.91
X3.ATEC2	0.79	0.62	0.62	0.87
X4.ATEC2	0.61	0.48	0.37	0.70
X5.ATEC2	0.78	0.61	0.60	0.84
X6.ATEC2	0.82	0.65	0.68	0.83
X7.ATEC2	0.89	0.70	0.80	0.81
X8.ATEC2	0.81	0.64	0.66	0.84
X9.ATEC2	0.76	0.60	0.58	0.80
X10.ATEC2	0.78	0.61	0.61	0.86
X11.ATEC2	0.89	0.70	0.78	0.88
X12.ATEC2	0.84	0.66	0.70	0.83
X13.ATEC2	0.84	0.66	0.70	0.76
X16.ATEC2	0.91	0.72	0.84	0.90
X17.ATEC2	0.87	0.69	0.76	0.90
X18.ATEC2	0.90	0.71	0.80	0.87
X19.ATEC2	0.86	0.67	0.73	0.90
X20.ATEC2	0.80	0.63	0.64	0.84

Kaiser–Meyer–Olkin Value = 0.85
Fit Based Upon Off-Diagonal Values = 0.95
Root Mean Square of the Residuals = 0.16
Sum of the Squared Loadings = 12.15
Proportion Variance = 67%
Cronbach's Alpha = 0.92 (95% CI: 0.90, 0.95)

Table S11: Exploratory Factor Analysis of ATEC Subscale 3 Cognitive Awareness

Items	Factor Loadings	Factor Score Correlations	Communality	Measure of Sampling Adequacy
X1.ATEC3	0.75	0.56	0.57	0.84
X2.ATEC3	0.81	0.61	0.66	0.83
X3.ATEC3	0.75	0.56	0.57	0.85
X4.ATEC3	0.63	0.47	0.40	0.77
X5.ATEC3	0.80	0.60	0.64	0.88
X6.ATEC3	0.92	0.69	0.84	0.90
X7.ATEC3	0.97	0.73	0.95	0.93
X8.ATEC3	0.83	0.62	0.68	0.83
X9.ATEC3	0.87	0.66	0.77	0.86
X10.ATEC3	0.94	0.70	0.88	0.91
X11.ATEC3	0.88	0.66	0.78	0.90
X12.ATEC3	0.93	0.70	0.86	0.90
X13.ATEC3	0.87	0.65	0.76	0.88
X14.ATEC3	0.73	0.55	0.54	0.83
X15.ATEC3	0.72	0.54	0.51	0.79
X16.ATEC3	0.68	0.51	0.46	0.85
X17.ATEC3	0.74	0.56	0.55	0.77
X18.ATEC3	0.93	0.69	0.86	0.91

Kaiser–Meyer–Olkin Value = 0.87
Fit Based Upon Off-Diagonal Values = 0.95
Root Mean Square of the Residuals = 0.16
Sum of the Squared Loadings = 12%
Proportion Variance = 68%
Cronbach's Alpha = 0.92 (95% CI: 0.89, 0.94)

Table S12: Exploratory Factor Analysis of ATEC Subscale 4 Health

Items	Factor Loadings	Factor Score Correlations	Communality	Measure of Sampling Adequacy
X1.ATEC4	0.64	0.50	0.40	0.74
X2.ATEC4	0.70	0.56	0.50	0.74
X3.ATEC4	0.69	0.55	0.48	0.70
**X4.ATEC4**	**0.55**	0.43	0.30	0.69
**X5.ATEC4**	**0.44**	0.35	0.19	0.48
X6.ATEC4	0.65	0.52	0.42	0.77
X7.ATEC4	0.71	0.56	0.51	0.64
X8.ATEC4	0.60	0.48	0.36	0.60
X9.ATEC4	0.65	0.51	0.42	0.63
**X10.ATEC4**	**0.17**	0.13	0.03	0.35
X11.ATEC4	0.68	0.54	0.47	0.69
X12.ATEC4	0.67	0.53	0.45	0.77
X13.ATEC4	0.85	0.68	0.73	0.78
X14.ATEC4	0.56	0.45	0.32	0.74
**X15.ATEC4**	**0.43**	0.34	0.18	0.54
**X16.ATEC4**	**0.23**	0.18	0.05	0.30
**X17.ATEC4**	**0.33**	0.26	0.11	0.53
X18.ATEC4	0.62	0.49	0.39	0.77
X19.ATEC4	0.56	0.44	0.31	0.61
X20.ATEC4	0.75	0.59	0.56	0.73
X21.ATEC4	0.59	0.47	0.35	0.57
X22.ATEC4	0.72	0.57	0.52	0.75
X23.ATEC4	0.62	0.49	0.39	0.62
X24.ATEC4	0.91	0.73	0.84	0.83
X25.ATEC4	0.60	0.48	0.36	0.66

Kaiser–Meyer–Olkin Value = 0.68
Fit Based Upon Off-Diagonal Values = 0.76
Root Mean Square of the Residuals = 0.22
Sum of the Squared Loadings = 9.62
Proportion Variance = 38%
Cronbach's Alpha = 0.88 (95% CI: 0.84, 0.91)

Table S13: Exploratory Factor Analysis of ATEC Subscale 4 Health after removing items 4, 5, 10, 15, 16, and 17

Items	Factor Loadings	Factor Score Correlations	Communality	Measure of Sampling Adequacy
X1.ATEC4	0.65	0.50	0.43	0.76
X2.ATEC4	0.70	0.54	0.50	0.73
X3.ATEC4	0.70	0.54	0.50	0.75
X6.ATEC4	0.73	0.56	0.54	0.83
X7.ATEC4	0.72	0.55	0.52	0.69
X8.ATEC4	0.60	0.46	0.36	0.57
X9.ATEC4	0.69	0.52	0.47	0.70
X11.ATEC4	0.73	0.56	0.54	0.79
X12.ATEC4	0.73	0.56	0.53	0.82
X13.ATEC4	0.90	0.68	0.80	0.84
X14.ATEC4	0.59	0.45	0.35	0.68
X18.ATEC4	0.63	0.48	0.40	0.63
X19.ATEC4	0.59	0.45	0.35	0.67
X20.ATEC4	0.81	0.61	0.65	0.78
X21.ATEC4	0.65	0.49	0.42	0.70
X22.ATEC4	0.76	0.58	0.58	0.78
X23.ATEC4	0.70	0.53	0.49	0.73
X24.ATEC4	0.95	0.72	0.90	0.85
X25.ATEC4	0.72	0.55	0.52	0.75

Root Mean Square of the Residuals = 0.21
Sum of the Squared Loadings = 9.84
Proportion Variance = 52%
Cronbach's Alpha = 0.88 (95% CI: 0.84, 0.92)

Table S14: Confirmatory Factor Analysis of ATEC Subscale 1 Expressive Language

Items	Standardized Factor Loadings	P-Value
X1.ATEC1	0.40	<0.0001
X2.ATEC1	0.54	0.003
X3.ATEC1	0.45	0.003
X4.ATEC1	0.58	0.003
X5.ATEC1	0.77	0.001
X6.ATEC1	0.84	0.002
X7.ATEC1	0.57	0.001
X8.ATEC1	0.85	0.003
X9.ATEC1	0.77	0.002
X10.ATEC1	0.82	0.003
X11.ATEC1	0.82	0.002
X12.ATEC1	0.76	0.004
X13.ATEC1	0.80	0.004
X14.ATEC1	0.70	0.005

Root Mean Square Error of Approximation = 0.048 (90% CI: 0.00, 0.082)
Comparative Ft Index = 0.994
Tucker–Lewis Index = 0.993
Standardized Root Mean Square Residual = 0.118

Table S15: Confirmatory Factor Analysis of ATEC Subscale 2 Sociability

Items	Standardized Factor Loadings	P-Value
X1.ATEC2	0.63	<0.0001
X2.ATEC2	0.83	<0.0001
X3.ATEC2	0.61	<0.0001
X4.ATEC2	0.41	<0.0001
X5.ATEC2	0.71	<0.0001
X6.ATEC2	0.75	<0.0001
X7.ATEC2	0.67	<0.0001
X8.ATEC2	0.58	<0.0001
X9.ATEC2	0.75	<0.0001
X10.ATEC2	0.40	<0.0001
X11.ATEC2	0.68	<0.0001
X12.ATEC2	0.67	<0.0001
X13.ATEC2	0.69	<0.0001
**X14.ATEC2**	0.16	0.151
**X15.ATEC2**	0.09	0.422
X16.ATEC2	0.72	<0.0001
X17.ATEC2	0.69	<0.0001
X18.ATEC2	0.70	<0.0001
X19.ATEC2	0.77	<0.0001
X20.ATEC2	0.53	<0.0001

Root Mean Square Error of Approximation = 0.075 (90% CI: 0.055, 0.094)
Comparative Ft Index = 0.873
Tucker–Lewis Index = 0.858
Standardized Root Mean Square Residual = 0.094

Table S16: Confirmatory Factor Analysis of ATEC Subscale 2 Sociability after removing items 14 and 15

Items	Standardized Factor Loadings	P-Value
X1.ATEC2	0.63	<0.0001
X2.ATEC2	0.83	<0.0001
X3.ATEC2	0.62	<0.0001
X4.ATEC2	0.39	0.0002
X5.ATEC2	0.71	<0.0001
X6.ATEC2	0.75	<0.0001
X7.ATEC2	0.68	<0.0001
X8.ATEC2	0.58	<0.0001
X9.ATEC2	0.76	<0.0001
X10.ATEC2	0.39	<0.0001
X11.ATEC2	0.68	<0.0001
X12.ATEC2	0.66	<0.0001
X13.ATEC2	0.69	<0.0001
X16.ATEC2	0.72	<0.0001
X17.ATEC2	0.70	<0.0001
X18.ATEC2	0.70	<0.0001
X19.ATEC2	0.77	<0.0001
X20.ATEC2	0.53	<0.0001

Root Mean Square Error of Approximation = 0.063 (90% CI: 0.036, 0.086)
Comparative Ft Index = 0.928
Tucker–Lewis Index = 0.918
Standardized Root Mean Square Residual = 0.08

Table S17: Confirmatory Factor Analysis of ATEC Subscale 3 Sensory Awareness

Items	Standardized Factor Loadings	P-Value
X1.ATEC3	0.27	<0.0001
X2.ATEC3	0.49	0.001
X3.ATEC3	0.71	0.006
X4.ATEC3	0.32	0.075
X5.ATEC3	0.47	0.013
X6.ATEC3	0.79	0.006
X7.ATEC3	0.54	0.026
X8.ATEC3	0.53	0.003
X9.ATEC3	0.52	0.003
X10.ATEC3	0.55	0.004
X11.ATEC3	0.66	0.009
X12.ATEC3	0.74	0.007
X13.ATEC3	0.77	0.007
X14.ATEC3	0.31	0.007
X15.ATEC3	0.66	0.023
X16.ATEC3	0.65	0.033
X17.ATEC3	0.40	0.020
X18.ATEC3	0.70	0.008

Root Mean Square Error of Approximation = 0.053 (90% CI: 0.018, 0.078)
Comparative Ft Index = 0.98
Tucker–Lewis Index = 0.977
Standardized Root Mean Square Residual = 0.11

Table S18: Confirmatory Factor Analysis of ATEC Subscale 4 Health

Items	Standardized Factor Loadings	P-Value
X1.ATEC4	0.43	<0.0001
X2.ATEC4	0.47	<0.0001
X3.ATEC4	0.35	<0.0001
**X4.ATEC4**	0.13	0.197
**X5.ATEC4**	0.10	0.418
X6.ATEC4	0.53	0.002
X7.ATEC4	0.53	0.002
X8.ATEC4	0.64	0.000
X9.ATEC4	0.26	0.047
**X10.ATEC4**	0.03	0.817
X11.ATEC4	0.43	0.019
X12.ATEC4	0.39	0.017
X13.ATEC4	0.34	0.018
X14.ATEC4	0.39	0.019
**X15.ATEC4**	0.18	0.194
**X16.ATEC4**	0.23	0.041
**X17.ATEC4**	0.07	0.157
X18.ATEC4	0.42	0.016
X19.ATEC4	0.46	0.007
X20.ATEC4	0.48	0.002
X21.ATEC4	0.52	0.002
X22.ATEC4	0.32	0.025
X23.ATEC4	0.64	0.000
X24.ATEC4	0.60	0.001
X25.ATEC4	0.51	0.001

Root Mean Square Error of Approximation = 0.060 (90% CI: 0.041, 0.077)
Comparative Ft Index = 0.887
Tucker–Lewis Index = 0.876
Standardized Root Mean Square Residual = 0.131

Table S19: Confirmatory Factor Analysis of ATEC Subscale 4 Health after removing items 4, 5, 10, 15, 16, and 17

Items	Standardized Factor Loadings	P-Value
X1.ATEC4	0.42	
X2.ATEC4	0.45	0.00
X3.ATEC4	0.34	0.00
X6.ATEC4	0.52	0.00
X7.ATEC4	0.52	0.00
X8.ATEC4	0.63	0.00
X9.ATEC4	0.28	0.04
X11.ATEC4	0.42	0.02
X12.ATEC4	0.39	0.02
X13.ATEC4	0.35	0.02
X14.ATEC4	0.38	0.02
X18.ATEC4	0.42	0.02
X19.ATEC4	0.47	0.01
X20.ATEC4	0.49	0.00
X21.ATEC4	0.52	0.00
X22.ATEC4	0.34	0.02
X23.ATEC4	0.64	0.00
X24.ATEC4	0.60	0.00
X25.ATEC4	0.51	0.00

Root Mean Square Error of Approximation = 0.067 (90% CI: 0.043, 0.088)
Comparative Ft Index = 0.915
Tucker–Lewis Index = 0.905
Standardized Root Mean Square Residual = 0.133
